# Biorhizome: A Biosynthetic Platform for Colchicine Biomanufacturing

**DOI:** 10.3389/fpls.2017.01137

**Published:** 2017-06-30

**Authors:** Ganapathy Sivakumar, Kamran Alba, Gregory C. Phillips

**Affiliations:** ^1^Department of Engineering Technology, College of Technology, University of Houston, HoustonTX, United States; ^2^College of Agriculture and Technology, Arkansas State University, JonesboroAR, United States

**Keywords:** anticancer, antigout, bioprocess, *Gloriosa superba*, transcriptome

## Abstract

Colchicine is one of the oldest plant-based medicines used to treat gout and one of the most important alkaloid-based antimitotic drugs with anticancer potential, which is commercially extracted from *Gloriosa superba*. Clinical trials suggest that colchicine medication could prevent atrial fibrillation recurrence after cardiac surgery. In addition, therapeutic colchicine is undergoing clinical trials to treat non-diabetic metabolic syndrome and diabetic nephropathy. However, the industrial-scale biomanufacturing of colchicine have not yet been established. Clearly, further studies on detailed biorhizome-specific transcriptome analysis, gene expression, and candidate gene validation are required before uncover the mechanism of colchicine biosynthesis and biorhizome-based colchicine biomanufacturing. Annotation of 32312 assembled multiple-tissues transcripts of *G. superba* represented 15088 unigenes in known plant specific gene ontology. This could help understanding colchicine biosynthesis in *G. superba*. This review highlights the biorhizomes, rhizome specific genes or gene what expressed with high level in rhizomes, and deep fluid dynamics in a bioreactor specifically for the biomanufacture of colchicine.

## Introduction

Alkaloids are one of the most chemically diverse nitrogenous small molecules which are synthesized from amino acids. Many bioactive alkaloids are extracted from plants which have been used for human medicine (Schläger and Dräger, 2016). The Colchicaceae family has a unique colchicine alkaloid biosynthetic mechanism (Chacón et al., 2014). *Gloriosa superba* L. is a member of Colchicaceae, and is a very successful commercial source of pharmaceutical colchicine (Sivakumar, 2013). Colchicine has several molecular functions (Kwon et al., 2017; Prins et al., 2017). First, colchicine has very strong binding affinity for tubulin that prevents the microtubule assembly and thereby inhibits cell division (Herdman et al., 2016). This antimitotic mechanism has been used in chemotherapy to prevent cancer cell growth (Johnson et al., 2017). In addition, colchicine enhances the interleukin-8 production which could inhibit the human pancreatic cancer (Yokoyama et al., 2017). However, the anticancer applications of colchicine have been limited due to high clinically acceptable concentrations (Lin et al., 2016). Colchicine has been successfully used in plant cytogenetics to double chromosome numbers. For instance, colchicine inhibits the formation of spindle fibers at anaphase, resulting in replicated homozygous chromosomes as in cabbage and broccoli (Yuan et al., 2015). Second, colchicine has been widely used for centuries to treat gout (Wilson and Saseen, 2016; Abhishek et al., 2017). Colchicine treatment could decrease systemic inflammation (Akodad et al., 2017). Indeed, colchicine had antifibrotic effects in diabetic nephropathy (Solak et al., 2017). Finally, clinical data suggested that colchicine treatment could inhibit cardiovascular diseases, among others (Frommeyer et al., 2017).

Medical studies indicated that patients administered with the dose of 0.6 mg colchicine per day would show plasma concentration after single dosing of approximately 2 ng/ml, which has been shown to promote gout inhibition, while 6 ng/ml is required to observe gastric cancer inhibition (Terkeltaub et al., 2010; Lin et al., 2016). Overdoses can have devastating consequences or toxicity (Medani and Wall, 2016). Notably, appropriate *G. superba* crude extract doses could prevent unintended contraindications which have been reported in traditional treatments (Capistrano et al., 2016; Kande Vidanalage et al., 2016). The pharmaceutical quality control NMR analysis of enantiomer and synthetic racemic mixture of colchicine has been recently reported (Menéndez-López et al., 2017). *G. superba* seed and field grown rhizomes contain a unique colchicine scaffold with a high concentration of colchicine, approximately 0.9 and 0.3%, respectively (Sivakumar, 2013). Therefore, public biosafety is important in field cultivation, handling, and processing to prevent accidental poisoning of workers. Despite colchicine being highly studied in the medical sector, little is known about the biosynthesis in plants and biosynthetic genes have not yet been identified. Due to lack of this knowledge, there has been limited success in increasing the yield of *G. superba* rhizomes. Nevertheless, stable high colchicine accumulation is challenging and the cultivation is labor-intensive, time consuming, and expensive (Vanitha and Manimalathi, 2013). Use of natural colchicine has been increasing substantially in the pharmaceutical industry, thus, alternative biomanufacturing platforms must be developed (Sivakumar, 2017).

**FIGURE 1:**
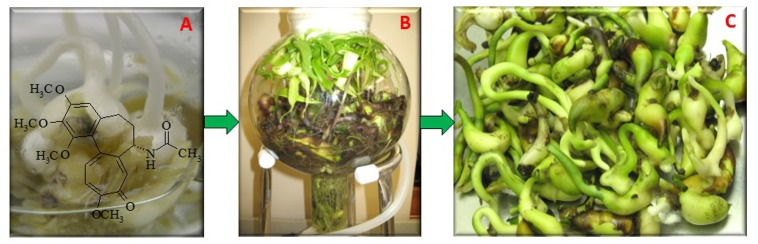
Illustration of workflow for *Gloriosa superba* biorhizome biomanufacturing. **(A)** Biorhizome induction from callus (50 ml flask) – the chemical structure is colchicine; **(B)** Biorhizome scaled-up in a 5 L airlift bioreactor (height: 16 inches; diameter 8 inches); **(C)** Harvested biorhizome from bioreactors.

Plant cell and root culture systems have been typically used in biotech industry to biomanufacture therapeutic molecules (Sivakumar et al., 2011; Tekoah et al., 2015). Despite considerable metabolic engineering or synthetic biotechnology efforts, the yield of bioactive alkaloid molecules are still very low in these systems because, in part, the lack of knowledge of the biosynthetic mechanism, pathways, and gene expression (Li and Smolke, 2016). *G. superba* and colchicum species root, callus and cell cultures have been conducted *in vitro*, but these cultures have yielded insignificant concentrations of colchicine (Daradkeh et al., 2012; Ghosh et al., 2015; Nikhila et al., 2017). Clearly, further advancement is needed to effectively overcome these barriers. Notably, *in vitro* bulbs are capable of producing montanine and hemanthamine alkaloids (Zayed et al., 2011). Since, rhizomatousness is one of the key lifecycle features in the perenniality of *G. superba*, the biorhizome can be used as an alternative colchicine production system. For instance, rhizomes are the predominant field propagation system for commercially grown *G. superba* (Phatak and Hegde, 2014; Padmapriya et al., 2015). Each *G. superba* daughter rhizome arises from a bifurcated mother rhizome, and each rhizome fork possesses one apical vegetative meristem (Mallya Suma et al., 2014). The apical rhizome buds are dynamic asexual organs which involve complex cross-talk between different regulatory levels, and grow into a complete plant which eventually becomes self-supporting (Salvato et al., 2015). There is very little gene expression information regarding rhizome development and cascade mechanisms involving biosynthesis of small molecules (Li et al., 2014). However, *G. superba in vitro* tuber cultures accumulate 0.01–0.1% DW of colchicine (Selvarasu and Kandhasamy, 2012; Kumar et al., 2015). Dormancy mechanisms may counteract biosynthesis of colchicine in field grown rhizomes, but this impediment has been overcome in the *G. superba* biorhizome. This review highlights new biotechnological biorhizome-based biomanufacturing to improve the therapeutic colchicine production in *G. superba* (**Figure 1**).

## Biorhizome

Biotechnological biorhizomes are asexually produced rootstocks grown *in vitro*, whose buds develop new shoots, adventitious roots, and daughter biorhizomes to serve as reproductive as well as storage organs (**Figure 1C**). They may be used to biosynthesize high-value pharmaceutical molecules. Biorhizomes are unique and efficient biosynthetic mechanisms in rhizomatous plants, and an advanced biotechnological platform compared to root and cell cultures (Sivakumar, 2017). Notably, the size of the shoot is directly related to the age and size of the biorhizome, perhaps because the rhizome is not only energy source but hormones source for the developing shoot (Winkel et al., 2011). The coordinating mechanism of the shoot and rhizome could balance the inorganic and organic carbon via photosynthesis and respiration, respectively (Sakamaki and Ino, 2006; Srinivasan et al., 2016). Biorhizomes continuously synthesize colchicine. This functional characteristic of continuous colchicine production is a decided advantage for biomanufacturing compared to root culture, in which colchicine production is quite low (Sivakumar, 2013). The biosynthesis of colchicine exploits the immobilization of the biosynthetic machinery within a differentiated specialized biorhizome.

At the molecular level, regulation of biorhizome formation is very complex but genes controlling shoot production might be involved (Balbuena et al., 2012; Kim et al., 2013). There is evidence that rhizome morphogenesis in Lotus is regulated by photoperiod (Hu et al., 2011; Cheng et al., 2013b). Hormone auxin are involved in the initiation and development of rhizomes in Lotus. Many genes exhibit significant changes in their expression during development, however, genes associated with auxin hormone signaling appear to trigger rhizome induction (Masuda et al., 2007; Cheng et al., 2013a; Novak and Whitehouse, 2013). In bamboo, about 26 genes are highly expressed in the rhizome buds, which are related to auxin biosynthesis and signaling. The transcriptional factor REVOLUTA was highly expressed in rhizome buds of bamboo, which plays an important role in meristem initiation (Wang et al., 2010). In potato, calmodulin-binding protein plays a regulatory role in signal transduction for tuber formation (Reddy et al., 2002). For instance, FT, Lov Kelch protein 2, CONSTAN, and GIGANTEA genes have been involved in the transduction of photoperiodic signals which might be promoting the rhizome budding in potato (Navarro et al., 2011; Yang et al., 2015). There were 14 other important rhizome formation-related genes, including a MADS-box that could be involved in rhizome enlargement (Cheng et al., 2013b). Genes encoding phytochrome B, CO, GI, and FT were identified in Lotus rhizomes, with differing gene expression and regulation in the shoot and rhizome (Yang et al., 2015). The transcription factor families such as AP2-EREBP, bHLH, MYB, NAC, and WRKY play an important role in regulating secondary metabolic pathways in rhizomes (Yang et al., 2012). In addition, miRNAs were differentially expressed in aerial shoots and rhizomes (Zonga et al., 2014). Thus, at the transcriptional level, shoots and biorhizomes are sharing the functional coordination.

Genomic and transcriptomic data generally suggest that gene transcripts involved in translation, transcription regulation, and metabolism were abundant in the rhizome, while in the leaf the gene transcripts for photosynthesis, stress response, and translation were the most dominant (Huang et al., 2016). Hence, the biorhizome is a unique system for identifying rhizome-specific genes for elucidating the colchicine pathway, and the biorhizome can be used as a biofactory to produce pharmaceutical colchicine. Interestingly, colchicine biosynthesis appears to be upregulated in the biorhizome relative to that in adventitious root culture. Gene expression patterns in the rhizome were quite diverse, while the primary and secondary metabolisms were upregulated (Chen and Li, 2016; Gurung et al., 2016). Apparently, the biorhizome biomass and the colchicine biosynthesis are interconnected with shoot production, but more colchicine was produced in the biorhizome than the shoot. For instance, the leaves and stems accumulate less than 0.1% colchicine whereas the biorhizome accumulate over 0.5% (DW) colchicine (Sivakumar, 2017). Indeed, the sprouts upregulate the colchicine production in the biorhizome. In bioreactor culture, the roots-detached biorhizome continuously grows and synthesizes colchicine, whereas shoots-detached biorhizome loses its function to synthesize biomass or colchicine. Despite this, metabolic adaptation or a gene network could enhance the translocation of colchicine from the shoots to the biorhizome, which is important for the plant’s survival.

Indeed, the shoots-detached biorhizome induces the new daughter biorhizome in bioreactor culture. This phenomenon suggests that shoots play a key molecular mechanisms in biorhizome and colchicine biosynthesis. This characteristic could be associated with changes in the fundamental expression pattern of genes, and alterations in various biochemical and physiological processes that would be crucial for growth and survival of biorhizomes. Genes involved in stress response were greatly upregulated in the rhizome (Yang et al., 2016). For instance, the rhizome encodes a mobile signaling protein, which could control the biorhizome formation (Lee et al., 2013). This suggests that biorhizome might have a complete set of the stress response pathway enzymes. In addition, increased levels of dissolved nutrients, oxygen and hormone in bioreactor culture could stimulate daughter biorhizome development. However, *G. superba* biorhizome transcriptome analysis and gene expression patterns need to be understood to ascertain and unravel the underlying biorhizome regulatory network.

## Transcriptome Analysis

**FIGURE 2:**
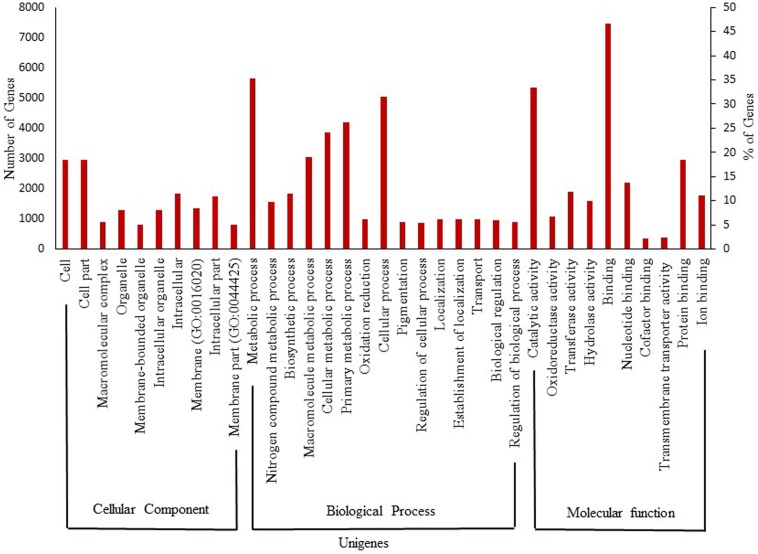
*Gloriosa superba* gene ontology classification of assembled unigenes.

The turmeric and ginger ESTs revealed that over 770 gene transcripts expressed in rhizomes, which are absent in other tissues. These transcripts were enriched for genes associated with rhizome development and regulation. The bioactive small molecules such as curcuminoids and gingerols synthesizing candidate genes were highly expressed in the rhizomes (Koo et al., 2013). Recently, deep sequencing transcriptome data was used to identify various unigenes involved in genome cellular component, biological process, molecular function, and proanthocyanidin biosynthesis in rhizome (Chen and Li, 2016). Notably, the benzylisoquinoline alkaloids biosynthetic genes were highly upregulated during bulb development in *Corydalis yanhusuo* (Liao et al., 2016). This suggests that rhizome has unique small molecule biosynthetic mechanism. However, there is no molecular information revealing the colchicine biosynthetic pathway in biorhizome. Advanced genomic, proteomic, metabolic, and bioprocess engineering efforts are required to overcome this barrier. Annotation of 32312 assembled transcript sequences, for multi-tissues including dormant rhizomes of *G. superba*, from the medicinal plant database^1^ represents 15088 unique genes having homology to known plant specific protein GO terms. For instance, in the cellular component domain, the terms cell (2795 genes, 18.5%, GO:0005623) and cell part (2795, 18.5%, GO:0044464) were mostly assigned. Within the biological function domain, the assignments were mostly enriched in the terms metabolic process (5306, 35.2%, GO:0008152) and cellular process (4746, 31.5%, GO:0009987). For the molecular function domain, the most evident matches were to the terms binding (7026, 46.6%, GO:0005488) and catalytic activity (5038, 33.4%, GO:0003824) (**Figure 2**). In addition, the *G. superba* transcriptome contains desired colchicine pathway candidate genes such as of *N*-methyltransferase, *O*-methyltransferases, P450s, and *N*-acetyltransferase (Sivakumar, 2017). Further studies on detailed biorhizome transcriptome analysis, gene expression, and candidate gene validation could uncover the mechanism of colchicine biosynthesis and development in *G. superba* biorhizomes, and facilitate metabolic engineering and industrial-scale biomanufacturing of colchicine.

## Biomanufacturing

Many human medicines are now biomanufactured by genetic engineering or recombinant DNA technology (Tekoah et al., 2015; Roh et al., 2016). Therapeutic small molecules with bioactive natural isomers are derived from biomanufacturing as part of a living system or cells (Sivakumar et al., 2006; Neville et al., 2017). The pharmaceutical quality control colchicine profile is important in raw plant tissue, necessitating that the colchicine molecule drug should not be altered. Therefore, the biomanufacturing is not only to transform a biorhizome system to produce therapeutic colchicine, but also to develop a safer production and quality control as mandated by regulatory agencies. Biomanufacturing colchicine from biorhizomes could lower upstream bioprocessing costs, incorporate economy of scale, speed production, reduce pesticide contamination of drugs.

**FIGURE 3:**
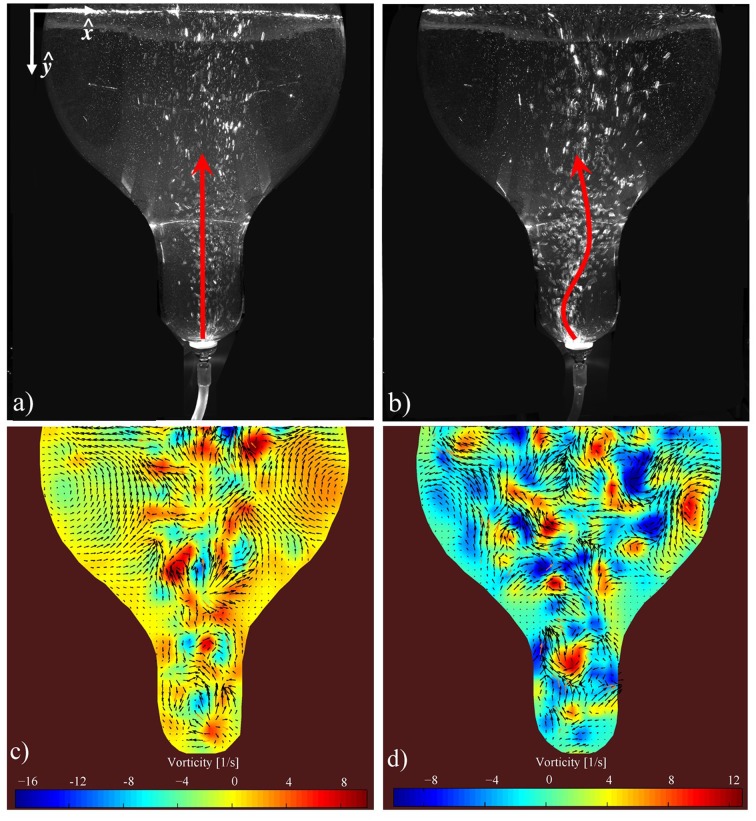
Multiphase flow mixing pattern in BTBR: **(a,b)** snapshots of air injection rates of 50 and 550 cc/min respectively within a 4-L working volume (water seeded with Polyamide Seeding Particles). The arrows indicate the direction of the ascending air bubbles **(a,b)**. **(c,d)** Corresponding Particle Image Velocimetry to **(a,b)**, respectively. The arrows represent the velocity field while the contours show the local vorticity intensity **(c,d)**.

Ginseng adventitious root culture has been successfully scaled-up in a BTBR (Sivakumar et al., 2005, 2011). Therefore, to scale-up *Gloriosa* biorhizome a BTBR has been used (**Figures 1B**, **3**). Successful biorhizome scale-up in BTBR require a deep fluid dynamics understanding, because the biorhizomes are completely immersed in the media. For instance, many engineering parameters are involved in the design of a BCR such as; gas density, 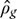, liquid density, 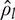, viscosity, 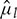, volumetric gas flow rate, 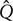, interfacial tension between gas and liquid phases, 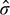, sparger pore size, 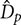, column diameter, 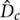, and length, 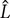. Such parameters will define mean diameter of the bubbles, 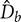, gas holdup (ratio of the gas phase to the total volume), ε, and superficial velocity defined as 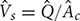 (Kantarci et al., 2005). Here, 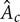 is the cross-sectional area of the column. The flow regimes in BCR are mainly classified according to the column diameter, 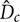, and the superficial gas velocity, 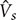.

Two types of flow regimes are commonly observed in BCR, namely *homogenous* (bubbly) and *heterogeneous* (churn-turbulent). A heterogeneous *slug* flow regime could also appear with small diameters at high gas flow rates. The bubbly flows, which can be either *perfect* or *imperfect* depending on the degree of the non-uniformity in bubble sizes that are usually obtained at low superficial gas velocities (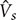 < 5 cm/s) (Bouaifi et al., 2001). The bubbles’ rising velocity and distribution in this regime is relatively steady, the mixing is gentle over the entire reactor and there is no bubble coalescence and/or break-up (Hua and Lou, 2007). Therefore, the bubble size is almost fully dictated by the sparger design and system properties (Ruzicka et al., 2001; Dhotre et al., 2004; Tang and Heindel, 2004; Thorat and Joshi, 2004). The gas holdup, ε, is found to increase linearly with superficial gas velocity, 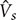. For higher gas injection rates (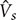 < 5 cm/s), churn-turbulent regimes are found, characterized by the coalescence/break-up of bubbles and increased turbulence and circulation (Hibiki and Ishii, 2000; Olmos et al., 2001; Buwa and Ranade, 2002; Michele and Hempel, 2002). This results in unsteady patterns and various bubble sizes ranging from a few millimeters to a few centimeters. Heat and mass transfer as well as liquid foaming may also introduce additional complexities (Lin and Wang, 2001; Cho et al., 2002; Li and Prakash, 2002; Chen et al., 2003; Krishna and Van Baten, 2003; Ruzicka and Thomas, 2003; Veera et al., 2004). Although several studies have identified the boundaries of possible BCR flow regimes, flow regimes in dimensionless maps have not been reported which is important for industrial design and scale-up. To generate dimensionless maps, the following Buckingham-π theorem analysis was used (Sopan Rahtika et al., 2017).

Following standard visualization techniques, the dynamics of the flow was characterized in a BTBR in the absence of nutrients and biorhizome to identify homogenous and heterogeneous regimes (Chen and Fan, 1992). The 5 L BTBR was used with 2 and 4 L working volume of polyamide seeding particles (PSP)-water solution at two different air injection rates (low injection rate 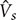 = 0.25 mm/s (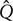 = 50 cm^3^/min) and higher injection rate 2.76 mm/s (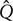 = 550 cm^3^/min) (**Figure 3**). **Figure 3a** suggests that in 4 L the air bubbles at low injection rate ascend up a fairly straight vertical path, concentrating mostly toward the center of the BTBR. However, at higher injection rates, a more chaotic flow forms (**Figure 3b**). In fact, upon leaving the sparger, the air bubbles oscillate in various directions over time. It is suggested that larger bubbles form at higher injection rate. **Figures 3c,d** show the 4 L velocity field corresponding to the experiments shown in **Figures 3a,b**, respectively. The formation of two major *vortices* are evident of the BTBR at low injection rate (**Figure 3c**). These major circulatory zones are disturbed (and thus shrunk) at higher flow rate (**Figure 3d**). The generated fluid mixing and circulation in a bioreactor can significantly affect the quality/quantity of the biorhizome biomass. In order to quantify the strength of the circulatory zones within the flow may calculate the *vorticity*, 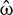, as 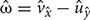, where 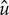 and 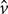 are the velocity components in 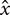 and 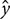 directions, respectively (**Figure 3a**). Here, 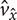 and 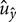 are simply the amount of flow shearing in 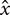 and 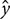 directions (Alba et al., 2014). The vorticity contours (in unit 1/s) have also been added to the velocity vectors shown in **Figures 3c,d** for comparison. The positive/negative values of the vorticity, 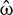, correspond to clockwise/counter-clockwise directions (**Figures 3c,d**). The positive and negative vorticity zones are propagated throughout a much larger BTBR domain at higher injection rate suggesting a more uniform mixing (**Figure 3d**). Both the strongest clockwise (positive 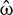) and counter-clockwise (negative 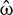) rotations were at higher injection rates. The 2 L flow pattern and dimensionless mapping are similar to 4 L. Further analysis is required to understand the counter-intuitive dynamics and flow regimes of such a complex system with biorhizome. Such flow analysis will not only be able to address the geometric patterns of mixing but extend to the nature of liquids, solutions, and injection gasses with various combinations of density, viscosity and surface tension that eventually will improve the biomanufacturing process design.

Critical culture conditions optimized in lab-scale (5–20 L) bioreactor for nutrients, temperature, and culture density may be emulated, at least in part, by that of colchicine biomanufacturing from biorhizomes. Workflow for *G. superba* upstream biomanufacturing has recently been reported for colchicine (Sivakumar, 2017). However, large-scale data and process validation are required for biorhizomes because during scale-up many working parameters inevitably differ from lab-scale to industrial-scale biomanufacturing. For instance, the nutrient utilization, oxygen level, convective media mixing, and growth factors become more challenging and airflow rate, shear stress profile, and mass transfer are significantly different from small- to large-scale (Roh et al., 2016). Moreover, maintaining reproducibility of biorhizome biomass and colchicine concentration requires homogenous microenvironmental parameters such as nutrients, oxygen, pH, and continuous removal of undesired molecules. These parameters should ideally be monitored online by automated computerized sensors, thereby standardizing the process control during the biomanufacturing processes, as has been done in industrial-scale bioreactors.

## Conclusion

Biomanufacturing utilizes the molecular mechanism of living systems and modifies their genome with upstream and downstream processes to develop efficient therapeutic products that help improve human health. Indeed, large-scale biomanufacturing of biopharmaceuticals is a rapidly growing sector of the bioeconomy. Biomanufacturing has utilized regulatory guidance to advance biopharmaceuticals for developing safe and effective medicine. The biorhizome has unique biosynthetic mechanism over plant cell or root cultures which could overcome small molecules production barriers in biomanufacturing. Moreover, biorhizome platforms could revolutionize colchicine upstream biomanufacturing, but first must resolve colchicine pathway elucidation challenges and biomass scale-up for the pharmaceutical industry. For cost-effective robust colchicine biomanufacturing, overproduction via metabolic engineering becomes an important upstream manufacturing step. Reprograming of colchicine biosynthetic pathway in biorhizome or synthetic biotechnology requires detailed pathway elucidation. While studies with large-scale airlift bioreactors for biorhizome manufacturing have not been conducted, a suitable model for colchicine biomanufacturing might be the industrial-scale process for ginsenosides biomanufacturing. More insight into the molecular mechanism of the biorhizome, its interactions with the shoot, as well as mass transfer are needed to fully understand and optimize the biosynthetic pathway for biomanufacturing of colchicine.

## Author Contributions

GS lead and designed the experiments and performed the biorhizome biomanufacturing, bioprocess engineering and analytical studies. GP helped bioreactor maintenance. KA performed the fluid mechanics.

## Conflict of Interest Statement

The authors declare that the research was conducted in the absence of any commercial or financial relationships that could be construed as a potential conflict of interest.

## Footnotes

1 http://www.medplantrnaseq.org

## Abbreviations

BCR: bubble column reactor
BTBR: balloon type bubble reactor
CO: CONSTAN
DW: dry weight
ESTs: expressed sequence tags
FDA: food and drug administration
FT: Flowering Locus T
GI: GIGANTEA
GO: gene ontology
KEGG: Kyoto encyclopedia of genes and genomes
NAT: *N*-acetyltransferase
NMR: nuclear magnetic resonance
NMT: *N*-methyltransferase
OMT: *O*-methyltransferases.
